# Interventions for domestic violence among pregnant women in low- and middle-income countries: a systematic review protocol

**DOI:** 10.1186/s13643-017-0657-6

**Published:** 2017-12-12

**Authors:** Diksha Sapkota, Kathleen Baird, Amornrat Saito, Debra Anderson

**Affiliations:** 1 School of Nursing and Midwifery, Griffith University, Brisbane, Australia; 2 Gold Coast University Hospital, Southport, QLD Australia; 3 Women’s Wellness Research Program, Menzies Health Institute Queensland, Brisbane, Australia; 4 Kathmandu University School of Medical Sciences, Dhulikhel, Nepal

**Keywords:** Domestic violence, Developing countries, Intervention studies, Pregnancy, Review

## Abstract

**Background:**

Violence during pregnancy is a global problem, associated with serious health risks for both the mother and baby. Evaluation of interventions targeted for reducing or controlling domestic violence (DV) is still in its infancy, and the majority of findings are primarily from high-income countries (HICs). Therefore, there is an urgent need for generating evidence of DV interventions among pregnant women in low- and middle-income countries (LMICs).

**Methods:**

Preferred Reporting Items for Systematic Reviews and Meta-Analyses (PRISMA) guidelines will be employed to structure the review. A comprehensive search will be carried out via electronic databases including MEDLINE, CINAHL, Scopus, Embase, Web of Science, PsycINFO, and The Cochrane library. Gray literature will also be scrutinized for potential articles. An optimal search strategy has been developed following consultations with subject-matter experts and librarians. This search strategy will be adapted to the different databases. Experimental studies evaluating DV interventions among pregnant women from LMICs will be included in the review. The review will only include literature written in English. Two reviewers will independently screen and assess studies for inclusion in the review. A third author will resolve any discrepancies between the reviewers. Risk of bias will be assessed based on the *Cochrane risk of bias assessment tool*, and overall quality of the evidence will be judged using Grading of Recommendations, Assessment, Development, and Evaluation (GRADE) criteria. Findings will be presented with the narrative synthesis, and if applicable, they will be further quantified using random-effects meta-analysis. Effect size, risk ratio for dichotomous variables, and standardized mean differences for continuous variables will be calculated for each outcome using Review Manager 5.3.

**Discussion:**

Systematic reviews to evaluate the efficacy of interventions to address DV within the perinatal context have been limited. Hence, no one intervention has emerged as substantially effective towards addressing perinatal DV and associated health consequences. The evidence generated from this systematic review will inform researchers and policy makers about the effectiveness of existing DV interventions among pregnant women in LMICs and provide recommendations for future research in this area. This in turn will contribute towards violence prevention in LMICs.

**Systematic review registration:**

PROSPERO CRD42017073938

**Electronic supplementary material:**

The online version of this article (10.1186/s13643-017-0657-6) contains supplementary material, which is available to authorized users.

## Background

### Statement of the problem

Violence against women (VAW) is a leading preventable contributor to death and disability and a significant economic burden [1]. Intimate partner violence (IPV) is the most common form of VAW and includes physical, sexual, and emotional abuse and controlling behaviors by an intimate partner [1, 2]. Globally, one in three women experiences violence from an intimate partner and one of the regions witnessing its highest prevalence is the South East Asian region (37.7%) [1]. Domestic violence (DV) is a term used in many countries, including low-and middle-income countries (LMICs), to refer to partner violence, but the term can also encompass child or elder abuse, or any other forms of abuse by any member of a household [2, 3]. In this review, domestic violence (DV) will be used to refer to any violence perpetrated against women by an intimate partner or by someone in her family relationship [2, 4].

Many women experience violence and abuse around the time of pregnancy [4, 5]. DV during pregnancy is more common than pre-eclampsia and placenta previa; notwithstanding this, it receives much less attention in perinatal care settings [5]. The WHO multi-country population survey, conducted in ten countries, identified the prevalence rate of DV during pregnancy ranging from 1% in urban Japan to 28% in provincial Peru [4]. A recent meta-analysis further supports its omnipresence reporting a higher proportion of victims from developing countries than developed countries (27.7 vs 13.3%) [6]. However, these prevalence rates are likely to be underestimated as they are based on self-reporting; with many women preferring to keep their history of violence in silence because of stigma, shame, and fear of retaliation [7].

DV during pregnancy is particularly alarming in light of its severe negative effects on the physical and mental health and well-being of both mother and child, and its overall effect on family functioning [8–10]. Evidence suggests DV during pregnancy is one of the strongest predictors for a wide array of mental health problems, such as anxiety, depression, post-traumatic stress disorder (PTSD) [11, 12], and persistent episodes of DV exerts soaring negative effects on mental health [13]. Similarly, almost one in every two women with mental illness is at risk of becoming a victim of DV [14, 15]. These figures support the reciprocal or cyclical relationship between violence and mental illness during pregnancy, both of which remain the major source of maternal morbidities [14]. It is unclear whether violence leads to mental illness or mental illness leads to violence or both morbidities coexist [3, 14, 16].

### Description of available interventions

Globally, various interventions have been developed for reducing DV and improving health outcomes. DV screening accompanied by comprehensive therapeutic intervention, such as counseling, psychotherapy, and home visiting, has shown some encouraging results [17–19]. Arroyo et al. concluded that the use of brief psychological treatments, such as cognitive behavioral therapy and psychoeducation, were found to increase the self-esteem and decrease the symptoms of depression and general distress among abused women [19]. The Cochrane review in 2015 assessed the effects of advocacy interventions, which included counseling, empathetic listening, and addressing the provision of social support, among women who have experienced abuse. The review concluded that though advocacy appeared to reduce violence and improve health outcomes, the magnitude and consistency of these benefits were uncertain [20]. Another systematic review conducted in 2015 reviewed 19 randomized controlled trials (RCTs) evaluating the effectiveness of home-visiting interventions in reducing partner violence. A home-visiting intervention, which included support services to women, was found effective in reducing DV in short term; however, there was no evidence that this change was sustained in the long term [21]. Nevertheless, regardless of the evidence base for interventions related to violence in the last decade, minimum research has stemmed from developing countries. Indeed, a disproportionately high number of the studies are based in high-income countries (HICs), and most of them do not consider DV in the context of pregnancy [17–21].

Pregnancy can be a critical turning point in a woman’s life [22]; some women may find it exceptionally stressful because of the physical, emotional, social, and economic changes in roles and needs that they may experience [23]. This may impede women’s coping skills, leading to an increased risk or escalation of domestic violence [22, 23]. Yet, at the same time, pregnancy presents a unique opportunity to identify victims and offer support to them because of repeated interactions with health care providers (HCPs) from early pregnancy to postpartum [5, 24]. The risk of abuse and the ability to access support services [3, 5] are entirely different for pregnant women; therefore, it is important to identify a tailored DV intervention which has the potential to meet the needs and expectations of pregnant women.

Jahanfar et al. [25] and Van Parys et al. [26] evaluated DV interventions around the time of pregnancy. Both reviews identified a number of DV interventions, including a single, brief, and individualized consultation to multiple therapy sessions both during pregnancy and after birth [25, 26]. Advocacy interventions consisting of supportive counseling, empathetic listening, and provision of social support were effective in reducing DV, depression, and other postpartum affective disorders [27, 28]. Home-visiting interventions aimed at enhancing maternal and child health and promoting healthy relationships were also found to reduce DV victimization in three studies [29–31]. Even so, due to a limited number of studies and lack of consistency in the outcomes, a meta-analysis could not be performed in either review [25, 26]. Consequently, both reviews were unable to provide a strong evidence base which would support the adoption of any intervention in the perinatal care context.

### Rationale for the current review

Even though RCTs are considered to be the gold standard for effectiveness studies [32], the ability to conduct such trials in LMICs are severely hampered by several logistical challenges [32, 33]. For example, in resource-constrained settings, there is a paucity of facilities, trained human resources, and expertise. Thus, the execution of randomized trials is hindered and limited by both high costs and time requirements [32, 33]. Furthermore, employing RCTs in complex public health interventions may be inappropriate, misleading, or unnecessarily expensive [33]. This may then partially explain why there are a minimal number of RCTs evaluating DV interventions in LMICs [25, 26]. However, having a small number of RCTs does not necessarily imply that prevention programs are not occurring in developing countries [8, 34], but to date, many of them may not have been employing a rigorous methodology [34]. A number of government and civil society organizations are now striving to develop interventions and resources to address DV in LMICs [34]. Indeed, a number of new interventions have been developed in recent years, but they have not been systematically assessed for comparative efficacy. Hence, this review will include a broader range of studies (both randomized controlled trials and non-randomized studies) to understand and incorporate a wider body of DV interventions that have been implemented and evaluated for pregnant women in LMICs.

The application of theory is advocated as an integral step in intervention design and evaluation and in evidence synthesis [35]. Theory-based behavior change interventions were found effective in various health promotion and disease prevention domains, such as IPV [31], HIV [36], and menopausal symptoms [37]. These interventions took into account the capacity and motivation of an individual to initiate and adhere to the change. Previous reviews have assessed the impact of DV interventions on several behavioral outcomes, such as safety planning, use of community resources, help-seeking behaviors, and self-efficacy, but, overall, they have not considered the theoretical underpinnings of the interventions [20, 25, 26]. The most important distinction of the current review will be the mapping of the multiple theoretical pathways to tackle DV among pregnant women. The review intends to provide a comprehensive description and illustration of how and why a desired change can be expected to occur in a particular context because of intervention.

WHO review asserts that the generalizability of results from HICs to LMICs cannot be assumed [8]. For example, a number of studies from HICs are currently using web-based interventions for behavioral changes in sensitive or stigmatized issues such as mental health and DV [38, 39]. Despite the effectiveness of such interventions, it is not possible to generalize such findings to all settings; this is particularly relevant to countries with limited resources and limited access to technology and the internet. For example, over 90% people from low-income countries (LICs) have no access to the internet [40] and computer literacy is generally low as well in these countries [41]. Hence, it must be recognized that an intervention that is successful in one context may not be applicable or exerts similar effects in another context [42, 43]. Several structural factors such as financial limitations, inadequate human resources, cultural barriers, social norms, and government policy may hinder the ability of low- and middle-income settings to deliver the interventions that have been successfully tested in HICs [44]. Therefore, there is a need to generate an evidence base from studies that can truly reflect the context of LMICs. Consequently, the main aim of this systematic review is to obtain a complete representation of intervention programs and their effectiveness in addressing DV and associated health consequences among pregnant women in resource-constrained settings.

### Review questions

- What are the effects of DV intervention on reducing the frequency and/or severity of DV among pregnant women in LMICs?
- What are the effects of DV interventions on secondary outcomes such as mental health, help-seeking behaviors, and use of community resources among the pregnant women in LMICs?
- What common theories have guided the design and/or implementation of DV interventions?

## Methods

The Preferred Reporting Items for Systematic Reviews and Meta-Analysis Protocols (PRISMA-P) recommendations [45] have been used for preparing and reporting this systematic review protocol (see Additional file 1). This systematic review protocol has been registered in the International Prospective Register of Systematic Reviews (PROSPERO) with registration number CRD42017073938.

### Search strategy

Medical Subject Headings (MeSH), controlled vocabulary, and key words have been used to identify articles. A combination of four key concepts is used “Domestic Violence,” “pregnant,” “LMICs,” and “intervention.” Boolean operators (“AND,” “OR,” and “NOT”) and proximity operators (“NEAR,” “NEXT,” and “ADJ”) have been used to combine search terms, and these operators will be adapted to the syntax of different databases. The Cochrane Handbook has been referred to identify the search terms for randomized and non-randomized study [46]. A comprehensive MEDLINE (Ovid) search strategy has been developed through an iterative approach in consultation with the health librarian and the review team [Additional file 2]. Other databases such as CINAHL, Web of Science, Scopus, Embase, The Cochrane Library (Cochrane Database of Systematic Reviews, Cochrane Central Register of Controlled Trials (CENTRAL)), and psycINFO will be searched with appropriate modification of MEDLINE strategy.

Several attempts will be made to search gray literatures in a systematic and transparent way. Google scholar, Cochrane Methodology Register, and WHO International Clinical trials registry will be searched for gray literatures presented as dissertations, abstracts, unpublished reports, and ongoing trials. Journals, such as *Journal of Interpersonal Violence*, *BMJ Injury Prevention*, *British Journal of Obstetrics and Gynecology*, *BMC Pregnancy and Childbirth*, *Journal of Family Violence*, *Violence against Women*, and *Journal of Women’s Health* will be systematically scrutinized for relevant literature. Reference lists and bibliography of the articles identified from the database searches will be cross-checked to ensure literature saturation.

### Inclusion criteria

Studies will be selected according to the PICOSS (participants/population, intervention, comparisons, outcomes, study designs, and settings) criteria outlined below:

- Participants: pregnant women of any age and/or women who have given birth in the past 12 months.
- Interventions: the study must have evaluated an intervention related to either DV or improving relationship or gender related issues. Intervention types may include, but not limited to, educational programs, group training, advocacy, empowerment, supportive counseling, referrals, home visitation, couple counseling, and other forms of DV-related intervention.
- Comparisons: either no intervention or usual care or standard care will be eligible.
- Outcomes: the primary outcome of this review will be self-reported measures of frequency and/or severity of DV (either physical, sexual, or emotional). Secondary outcomes will include the following:
  - Changes in psychological or mental health outcomes such as measures of quality of life, depression, anxiety, stress, self-efficacy, and self-esteem.
  - Use of safety behaviors, access to community resources, social support, and use of referral services.
- Studies: randomized controlled trials (RCTs) and non-randomized trials will be included. Non-randomized studies (NRS) will include non-randomized controlled trials (nRCT), controlled before and after (CBA) studies, and interrupted time series (ITS) studies.
- Settings: studies conducted in LMICs as listed by World Bank 2017 [47] will be included.

#### Additional inclusion criteria

- Published and unpublished articles written in English will be included. Reviewing published articles only may be subjected to publication bias, wherein positive results are over-emphasized due to the tendency for null or negative results not to get published [46]. Study protocols and conference abstracts will only be included if they contain pilot or preliminary results from the study whose data are otherwise unavailable.
- Multiple papers from a single study will be considered together and counted as one study. The paper containing the most comprehensive information will be included in data synthesis. However, if other papers report on any additional data or used different analytical methods across different papers, then, additional data will also be included.

### Exclusion criteria

- Any qualitative investigations, book chapters, case reports, letters, opinions, and editorials, will be excluded.
- Cohort studies, case–control studies, cross-sectional studies, will not be included.
- Studies conducted in HICs including women from LMICs will be excluded.

### Study selection

**Fig. 1:**
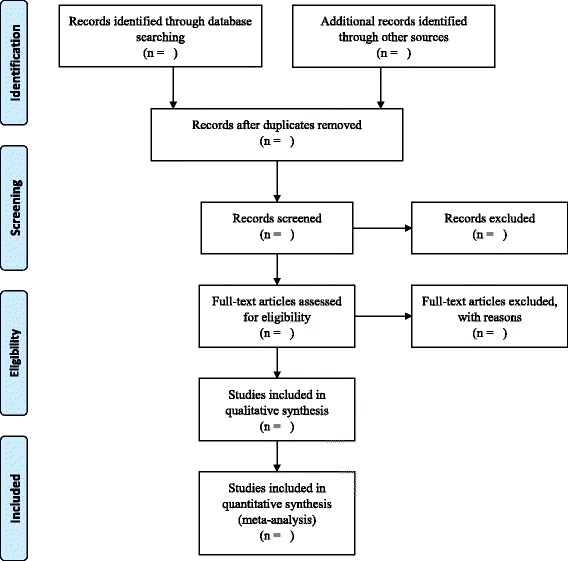
PRISMA flow diagram

The bibliographic software program Endnote (V.X8) will be used to manage and store relevant studies. Duplicate references will be removed via this software. Electronic searches will be scrutinized by two independent reviewers for eligibility and inclusion of studies into the review based on their title and abstract. This selection process will be piloted using 10% of papers and agreement between reviewers (DS and AS) will be assessed. When a difference of opinion occurs, the issue will be resolved with consensus involving a third reviewer (KB). Full text of potentially relevant articles will be retrieved and reviewed independently by two reviewers. A final inclusion or exclusion decision will be made on examination of full article, and reasons for exclusion will be documented for each excluded study. Figure 1 presents the flow diagram to be adopted in the systematic review for selecting the studies [48].

### Methodological appraisal of study

Classification of risk of bias as recommended by the Cochrane handbook will be used to assess the quality of selected RCTs. Main domains of bias assessment are sequence generation, allocation concealment, blinding (research personnel, participants, and outcome assessment), incomplete outcome data, selective reporting, and other potential sources of bias. Each domain will be categorized as low risk, high risk, and “unclear” if there will be an unclear or unknown risk due to insufficient information or lack of relevance [46]. For the non-randomized studies, Risk of Bias in Non-randomized Studies − of Interventions (ROBINS-I) assessment tool will be used [46].

### Data extraction

Study findings will be extracted on a structured database, developed in consultation with the research team. It includes pertinent information such as study setting, study population, sample size, study population’s demographics, baseline measurements, details related to intervention and control conditions, theories used, study methodology, response rates, follow-up, outcomes, times of measurement, and assessment of risk of bias (see Additional file 3). Data from individual studies will be extracted by DS using a piloted extraction form and then independently checked by AS. Discrepancies will be resolved by discussion and, where necessary, KB will make the final judgment. New categories will be added and extraction database will be modified as needed. Authors will be contacted to supply missing data or other relevant information if needed.

#### Timing of outcome assessment

As there is no optimal time for follow-up, the duration of follow-up in all studies will be documented. For the purpose of this review, follow-up of up to 6 months after the intervention will be considered short-term and follow-up after the 6 months will be categorized as long-term follow-up.

### Data analysis

A narrative synthesis of the findings from the included studies will be presented. The narrative synthesis will focus on socio-demographic characteristics of the study population, characteristics (study designs, settings, and sample size) of the studies included, details of the interventions (type, content, duration, intensity, and theories of change), effectiveness of the interventions, and pre-defined outcome measures.

#### Measurement of intervention effects

For RCTs, NRCTs, and CBA studies, dichotomous outcomes will be presented as risk ratio (RR), and if adjusted analyses reported dichotomous outcomes (adjusting for potential confounders) in terms of odds ratio (OR), Review Manager (RevMan) 5.3 will be used to convert ORs to RRs [49]. Anticipating that the included studies might have used a variety of measures to assess the same outcome, standardized mean difference (SMD) will be calculated for continuous outcomes.

For ITS studies, changes in the level of outcome (extrapolating pre-intervention regression line to first point post-intervention) and changes in trend (post-intervention regression slope minus pre-intervention regression slope) as determined by the included studies will be used.

#### Subgroup analysis and assessment of heterogeneity

Clinical heterogeneity will be assessed by examining the characteristics of studies and similarities between the types of participants and the interventions. Statistical heterogeneity will be assessed by calculating *I* ^2^ value. An *I* ^2^ value greater than 50% will be considered as indicative of substantial heterogeneity. If sufficient information is available from the included studies, subgroup analysis will be performed to check if the intervention effect varies with the study design and intervention characteristics (timing, duration, and intensity of the intervention). Providing there is an adequate number of studies (*n* ≥ 10) [46], meta-regression will be performed to identify between-study heterogeneity (in terms of duration of intervention and follow-up time).

#### Assessment of reporting bias

In case of appropriate number of studies (*n* ≥ 10), publication bias will be assessed by funnel plots for each outcome by plotting the effect size against study size [46].

#### Data synthesis

Results from different study designs will not be pooled together (for example, RCTs and NRS) to prevent a misleading summary of the study effect [46]; rather, they will be analyzed separately. When two or more studies are sufficiently homogeneous and comparable across the interventions, study designs, and outcomes measured, random-effects model will be utilized for meta-analysis, as it considers heterogeneity (within-study variance and between-study variance) in the effect estimate [46]. RevMan 5.3 will be used for statistical calculation [49]. Statistical differences for pooled SMD (for continuous variables) will be assessed using *Z* test at 0.05 level of significance. Similarly, for dichotomous data, the Mantel-Haenszel method will be used to calculate RR and effect sizes will be reported along with their 95% confidence interval (CI). If the studies are methodologically diverse, the findings will be presented descriptively only.

GRADE criteria will be used to assess the quality of the evidence for each outcome as recommended in the *Cochrane Handbook for Systematic Reviews of Interventions* [46]. The quality of evidence will be rated as high, moderate, low, and very low, and the factor that may decrease the quality of the evidence are study design, risk of bias, inconsistency of results, indirectness (not generalizable), imprecision (sparse data), and publication bias [46]. The reason for downgrading the quality of evidence will be provided. Summary of the findings will be presented in a table, including pre-specified outcomes, effect measures, number of studies and participants, and grade of overall quality of the evidence.

## Discussion

This protocol states the plan for a systematic review and meta-analysis of effectiveness of DV interventions among pregnant women in LMICs. Previous reviews were unable to provide a firm conclusion that a specific intervention was effective for addressing DV during pregnancy. This review will carry out a thorough search of the existing evidence for continued progress made, as well as an evaluation of the quality of the evidence of DV interventions available in LMICs. Whichever interventions service providers choose must be evidence-based and contextual. However, it can be seen that the evidence-base for prevention programs is over representative of developed countries in most cases. Therefore, this review will fill this gap by generating an evidence-base on effectiveness of different approaches for violence prevention and management targeted for pregnant women in resource-constrained settings. The findings could also point to the components, modes of delivery, and theoretical underpinnings of interventions which can serve as valuable inputs for future research in this area.

## Additional files

Additional file 1: PRISMA-P checklist. PRISMA-P (Preferred Reporting Items for Systematic review and Meta-Analysis Protocols) 2015 checklist. (DOCX 24 kb)

Additional file 2: MEDLINE search strategy. Strategy used to search articles in MEDLINE (Ovid) database. (DOCX 21 kb)

Additional file 3: Data items. Data items included the items that will be considered while extracting the data from included studies. It will be used to create structured database. (DOCX 21 kb)

## Abbreviations

CBA: Controlled before and after
CI: Confidence interval
DV: Domestic violence
HICs: High-income countries
IPV: Intimate partner violence
ITS: Interrupted time series
LMICs: Low- and middle-income countries
MeSH: Medical Subject Headings
nRCT: Non-randomized controlled trials
NRS: Non-randomized studies
PRISMA: Preferred Reporting Items for Systematic Review and Meta-analyses
PRISMA-P: Preferred Reporting Items for Systematic Review and Meta-Analysis Protocols
PTSD: Post-traumatic stress disorder
RCTs: Randomized controlled trials
VAW: Violence against women
WHO: World Health Organization
