# Supplementary material for: Interventions for domestic violence among pregnant women in low- and middle-income countries: a systematic review protocol
Source: Syst Rev. 2017 Dec 12;6:254. doi: 10.1186/s13643-017-0657-6 (PMC5727941; doi:10.1186/s13643-017-0657-6)
Supplement: Supplementary file 2 — MEDLINE search strategy. Strategy used to search articles in MEDLINE (Ovid) database. (DOCX 21 kb) [file 13643_2017_657_MOESM2_ESM.docx]

**Medline Search Strategy**

| **S.N.** | **Search terms** | **Identified results** |
| --- | --- | --- |
| 1 | exp Domestic Violence/ | 42335 |
| 2 | Child Abuse/ | 20901 |
| 3 | Elder Abuse/ | 2333 |
| 4 | 1 not (2 or 3) | 19234 |
| 5 | Battered Women/ | 2631 |
| 6 | exp Intimate Partner Violence/ | 8287 |
| 7 | ((domestic or relationship or partner* or family or families or spouse or wom#n or female* or wife or wives or girlfriend*) adj (abus* or violen* or beat* or batter*)).mp. | 16305 |
| 8 | ((physical or emotional or sexual or psychological or dating) adj (abus* or violence)).mp | 17625 |
| 9 | ((gender or intimate) adj1 violence) | 335 |
| 10 | Pregnant Women/ | 6614 |
| 11 | *Pregnancy/ | 33000 |
| 12 | ((pregnant or expect* or childbearing) adj1 (mother? or wom?n)).mp. | 85871 |
| 13 | randomized controlled trial/ | 496627 |
| 14 | exp Randomized Controlled Trials as Topic/ | 124035 |
| 15 | controlled clinical trial/ | 99233 |
| 16 | Controlled Before-After Studies/ | 297 |
| 17 | Interrupted Time Series Analysis/ | 366 |
| 18 | Non-Randomized Controlled Trials as Topic/ | 253 |
| 19 | evaluation studies/ | 242571 |
| 20 | "Outcome Assessment (Health Care)"/ | 66499 |
| 21 | Program Evaluation/ | 58335 |
| 22 | Nursing Evaluation Research/ | 10393 |
| 23 | "Early Intervention (Education)"/ | 2570 |
| 24 | Crisis Intervention/ | 5745 |
| 25 | (((((intervention? or evaluation) adj1 (study or trial or design?)) or ("quasi-experiment*" or "quasi random*" or "quasi control*" or (quasi or experimental))) adj1 (method? or stud* or trial? or design?)) or "randomi?ed controlled trial" or "controlled clinical trial" or "controlled before-after stud*" or "pre-post test" or intervention? or "intervention stud*" or program? or trial? or evaluation or "evaluation stud*" or "health promotion" or education or training or screen* or prevent* or testing or protocol or impact? or outcome? or control or assessment or output? or treatment or advocacy or counsel* or therapy).mp. | 11223910 |
| 26 | or/ 13-25 | 11223956 |
| 27 | animals/ not humans/ | 4642862 |
| 28 | 26 not 27 | 9603217 |
| 29 | (((developing or underserved or less developed or underdeveloped) adj1 (countr* or nation? or population)) or (('low income' or 'middle income') adj1 (countr* or nation? or population or women)) or ((transitional or poor or poorer) adj1 (countr* or nation? or population or world)) or 'low income' or 'middle income' or lic or lmic or africa or asia or caribbean or 'West Indies' or 'South America' or 'Latin America' or 'Central America' or 'Sub sahara' or saharan or 'South Africa').mp. | 325198 |
| 30 | (afghanistan or albania or algeria or "American Samoa" or angola or argentina or armenia or armenian or azerbaijan or bangladesh or byelarus or byelorussian or belarus or belorussian or belorussia or belize or benin or bhutan or bolivia or "Bosnia and Herzegovina" or bosnia or hercegovina or botswana or brasil or brazil or bulgaria or "Burkina Faso" or "Burkina Fasso" or burundi or "Cabo Verde" or cambodia or cameroon or cameron or camerons or "Central African Republic" or chad or china or colombia or comoros or "Comoro Islands" or comores or "Democratic Republic of Congo" or "Republic of the Congo" or congo or "Costa Rica" or "Cote d'Ivoire" or "Ivory Coast" or cuba).mp. | 371075 |
| 31 | (djibouti or "French Somaliland" or dominica or "Dominican Republic" or ecuador or egypt or "United Arab Republic" or "El Salvador" or "Equatorial Guinea" or eritrea or ethiopia or fiji or gabon or "Gabonese Republic" or gambia or georgia or georgian or ghana or grenada or guatemala or guinea or guiana or "Guinea Bissau" or guyana or haiti or honduras or india or indonesia or iran or "Islamic republic" or iraq or jamaica or jordan or kazakhstan or kazakh or kenya or kiribati or korea or kosovo or kyrgyzstan or kirghizia or "Kyrgyz Republic" or kirghiz or kirgizstan or "Lao PDR" or laos or lebanon or lesotho or liberia or libya).mp. | 479810 |
| 32 | (macedonia or madagascar or "Malagasy Republic" or malawi or malaysia or malaya or maldives or mali or "Marshall Islands" or mauritania or mauritius or "Agalega Islands" or mexico or micronesia or moldova or moldovia or moldovian or mongolia or montenegro or morocco or ifni or mozambique or myanmar or myanma or burma or namibia or nepal or nicaragua or niger or nigeria or pakistan or palau or panama OR "Papua New Guinea" or paraguay or peru or philippines or philipines or phillipines or phillippines).mp. | 192455 |
| 33 | (romania or rumania or roumania or russia or russian or "russian federation" or rwanda or ruanda or samoa or "Samoan Islands" or "Navigator Island" or "Navigator Islands" or "Sao Tome" or "sao tome and Principe" or senegal or serbia or "Sierra Leone" or "Solomon Islands" or somalia or "South Sudan" or "South Africa" or "Sri Lanka" or " St. Lucia" or "St Vincent" or "Grenadines" or sudan or suriname or surinam or swaziland or syria or "Syrian Arab Republic" or tajikistan or tadzhikistan or tadjikistan or tadzhik or tanzania or thailand or "timor-leste" or "timor" or togo or "Togolese Republic" or tonga or tunisia or turkey or turkmenistan or turkmen or tuvalu or uganda or ukraine or uruguay or ussr or "Soviet Union" or "Union of Soviet Socialist Republics" or uzbekistan or uzbek or vanuatu or "New Hebrides" or venezuela or vietnam or "Viet Nam" or "West Bank" or gaza or yemen or yugoslavia or zambia or zimbabwe).mp. | 356097 |
| 34 | or/ 29-33 | 1461260 |
| 35 | or/ 10-12 | 110461 |
| 36 | or/ 4-9 | 33857 |
| 37 | 35 and 36 | 911 |
| 38 | 34 and 37 | 348 |
| 39 | 28 and 38 | 303 |
| 40 | (comment or letter or editorial or news or newspaper article or case report).pt. | 1729322 |
| 41 | 39 not 40 | 303 |
| 42 | limit 41 to English language | 294 |
