# Supplementary material for: Interventions for domestic violence among pregnant women in low- and middle-income countries: a systematic review protocol
Source: Syst Rev. 2017 Dec 12;6:254. doi: 10.1186/s13643-017-0657-6 (PMC5727941; doi:10.1186/s13643-017-0657-6)
Supplement: Supplementary file 3 — Data items. Data items included the items that will be considered while extracting the data from included studies. It will be used to create structured database. (DOCX 21 kb) [file 13643_2017_657_MOESM3_ESM.docx]

**Additional File 3: Data items**

| **Headings** | **S.N.** | **Sub headings** |
| --- | --- | --- |
| 1. Characteristics of the study | 1. | Study ID number |
|  | 2. | Author |
|  | 3. | Year of Publication |
|  | 4. | Journal |
|  | 6. | Setting, Country |
|  | 7. | Title |
|  | 8. | Study designs |
|  | 9. | Sample size |
|  | 10. | Period |
|  | 11. | Follow up |
| 1. Socio-demographic characteristics of study population | 1. | Age |
|  | 2. | Trimester of pregnancy |
|  | 3. | Place of residence |
|  | 4. | Socioeconomic status (education, occupation, income) |
|  | 5. | Ethnicity |
|  | 6. | No. of children |
|  | 7. | ANC visit |
|  | 8. | Behavioural factors (smoking, alcohol and other drugs) |
|  | 9. | Pre-existing medical conditions |
| 1. Information about the intervention | 1. | Setting where intervention delivered |
|  | 2. | Person delivering intervention |
|  | 3. | Intervention content |
|  | 4. | Session |
|  | 5. | Follow Up |
|  | 6. | Response rate |
| 1. Mental health characteristics | 1. | Self-reported prevalence of depression (antenatal, postnatal) |
|  | 2. | Self-reported prevalence of PTSD (antenatal, postnatal) |
|  | 3. | Self-reported prevalence of anxiety (antenatal, postnatal) |
|  | 4. | Self-reported mental health score |
|  | 5. | Quality of life |
|  | 6. | Perceived family/social support |
|  | 7. | Use of safety planning and behaviours |
|  | 8. | Perceived stress |
| 1. Domestic violence characteristics | 1. | Rates |
|  | 2. | Types (physical violence, sexual violence, emotional violence) |
|  | 3. | Others if any |
| 1. Gaps identified in the study | | |
| 1. Strengths of the study | | |
| 1. Assessment of risk of bias (on 6 domains as given by Cochrane Risk of Bias Tool) | | |
